# The Role of MeCP2 in Regulating Synaptic Plasticity in the Context of Stress and Depression

**DOI:** 10.3390/cells11040748

**Published:** 2022-02-21

**Authors:** Carla L. Sánchez-Lafuente, Lisa E. Kalynchuk, Hector J. Caruncho, Juan Ausió

**Affiliations:** 1 Division of Medical Sciences, University of Victoria, Victoria, BC V8W 2Y2, Canada; carlaliria@uvic.ca (C.L.S.-L.); lkalynchuk@uvic.ca (L.E.K.); hectorjcaruncho@uvic.ca (H.J.C.); 2 Department of Biochemistry and Microbiology, University of Victoria, Victoria, BC V8W 3P6, Canada

**Keywords:** methyl-CpG-binding protein 2 (MeCP2), epigenetics, reelin, depression, stress, synaptic plasticity, brain-derived neurotrophic factor (BDNF), transcriptional repression

## Abstract

Methyl-CpG-binding protein 2 (MeCP2) is a transcriptional regulator that is highly abundant in the brain. It binds to methylated genomic DNA to regulate a range of physiological functions implicated in neuronal development and adult synaptic plasticity. MeCP2 has mainly been studied for its role in neurodevelopmental disorders, but alterations in MeCP2 are also present in stress-related disorders such as major depression. Impairments in both stress regulation and synaptic plasticity are associated with depression, but the specific mechanisms underlying these changes have not been identified. Here, we review the interplay between stress, synaptic plasticity, and MeCP2. We focus our attention on the transcriptional regulation of important neuronal plasticity genes such as BDNF and reelin (RELN). Moreover, we provide evidence from recent studies showing a link between chronic stress-induced depressive symptoms and dysregulation of MeCP2 expression, underscoring the role of this protein in stress-related pathology. We conclude that MeCP2 is a promising target for the development of novel, more efficacious therapeutics for the treatment of stress-related disorders such as depression.

## 1. Chromatin and Epigenetics: A Brief Overview

In the eukaryote cell, genomic DNA is organized as a nucleoprotein complex in which approximately 200 bp of DNA wraps in two left-handed superhelical turns about a heterotypic histone octamer core comprising two H2A, H2B, H3, and H4 core histones. These repetitive discrete nucleoprotein subunits connect to each other by linker DNA domains (ranging from 20–60 bp), to which linker histones (histone H1) are bound. These complexes are referred to as nucleosomes [1,2,3,4]. They form a repetitive structure (beads on a string) that results in chromatin fiber [4]. The winged-helix domain (WHD) of histone H1 [5] also binds close to the entry and exit sites of DNA in the nucleosome [6,7,8], and this binding plays a critical role in the modulation of the chromatin fiber folding [9]. Both ‘core histones’ and ‘linker histones’ consist of a bipartite/tripartite protein organization in which an N-terminal intrinsically disordered domain (IDD) [10] flanks a structured domain WHD [11] for linker histones or a histone fold domain (HFD) [12] for core histones. Histones H2A, H2B, and histone H1 contain an additional C-terminal IDD. IDDs are a critical site for histone post-translational modifications.

The notion that postsynthetic chemical modifications of histones and DNA could alter gene expression arose as early as 1964 with the seminal observation made by Vincent Allfrey about the potential involvement of histone acetylation and methylation post-translational modifications (PTMs) in the regulation of RNA synthesis [13]. The specific sites and/or the lysine/arginine residues affected in each of the histones were unknown at the time.

In 1983, Feinberg and Vogelstein reported that DNA (gene) hypomethylation could distinguish several human cancers from their normal counterparts [14], indicating an important role of DNA methylation in the gene dysregulation observed in these cancers. In these cases, however, it was not difficult to identify the DNA base affected, as it was believed that 5-methylcytosine (m5C) within a CpG dinucleotide sequence was the only residue methylated in eukaryotes [15,16]. At about the same time, studies on human γ-globin gene expression provided direct evidence for the role of CpG methylation in the regulation of gene expression—namely, that methylation was shown to have a repressive effect [17]. The relevance of CpG methylation to gene expression prompted the search for protein readers of this marker, and soon, the methyl-CpG-binding protein 1 (MeCP1) was discovered [18], as well as several other members of the methyl-binding domain (MBD) family of proteins [19], including MeCP2 [20].

In 2000, Strahl and Allis put forward the histone code hypothesis, according to which, PTMs (acetylation, methylation, phosphorylation, etc.) occur at discrete, well-defined residues, and their combinations generate a code that could be read by functional protein effectors (readers). This provides a plethora of markers (more than 16 histone PTMs have been described to date) and combinations of markers with enormous potential for gene regulation that surpasses by orders of magnitude the 5 mC DNA potential. Moreover, although histone PTMs have a highly pervasive distribution in eukaryotes, the extent and function of DNA methylation vary extensively among different organisms within this group [21,22].

In 2005, Esteller’s group clearly showed that lifetime differences between monozygotic human twins are the result of epigenetic changes in DNA methylation and histone acetylation that affect the landscape of their gene expression. It was subsequently shown that such epigenetic molecular drivers could have long-term functional implications. For example, it is now clear that the quality of maternal postnatal care has specific epigenetic effects (DNA methylation, histone methylation) on gene expression within the brain hippocampal region in rats, leading to lasting functional changes in learning, memory, anxiety, and stress regulation [23].

DNA methylation and histone PTMs constitute the main epigenetic markers that are established by a dynamic set of highly specific enzymes (writers) with downstream effects elicited by equally specific individual proteins and/or protein complexes (readers), and they can be removed by enzymatic complexes (erasers) [24,25].

## 2. The Unique Epigenetics of the Brain

The brain has a set of “exceptional and unique epigenetic” players [26]. At the DNA methylation level, the brain is heavily methylated [27]. Not surprisingly, methyl MeCP2 levels are extremely high in the brain [28]. In cortical neurons, there is about one molecule of MeCP2 for every one to two nucleosomes [29], and hence, it is a unique constitutive chromatin protein of these cells.

A rather unique noncanonical DNA methylation component of the brain is the occurrence of mCH [H = adenine (A), C, or thymine (T)] particularly mCpA, which is present after birth [27]. This DNA methylation plays a very important role in the regulation of gene expression especially in MeCP2 regulated genes [30,31], as MeCP2 is the only member of the MBD family of proteins that selectively binds mCpA and its repeats [32]. Hence, mCH and MeCP2 are two distinct epigenetic regulators of the brain.

MeCP2 has a 60% unstructured organization [33], and hence, it constitutes a genuine representative of the family of intrinsically disordered proteins [10,34]. The protein has two isoforms MeCP2-E1 (498 amino acids) and -E2 (486 amino acids) that are the product of alternative splicing and differ in their 21 first N-terminal amino acids. Despite such small structural differences, the two isoforms exhibit unique structural and functional characteristics, as well as distinct DNA targeting sites [35], where the mCpA-repeat-binding recognition observed in the brain can most likely be attributed to the E1 isoform, the most abundant isoform of MeCP2 in this tissue.

The levels of MeCP2 in the brain change during the circadian cycle (Paz et al., 2015), and alterations affecting this cycle can have a subtle influence on the regulation of gene expression. Considering this and the high abundance of MeCP2 in neurons, the important role of this protein in both normal and altered functions of the brain is not surprising [36].

There is an epigenetic uniqueness that transcends DNA methylation and its readers. Quite recently, evidence has been presented for a new class of histone modifications, serotonylation of glutamine, at position 5 (Q5ser) on histone H3 tri-methylated at lysine 4 (H3K4me3), [H3K4me3Q5ser] [37]. Furthermore, in the adult brain, the histone variant H3.3 (which accumulates with aging) corresponds to over 93% of the total H3 pool [38].

**Figure 1:**
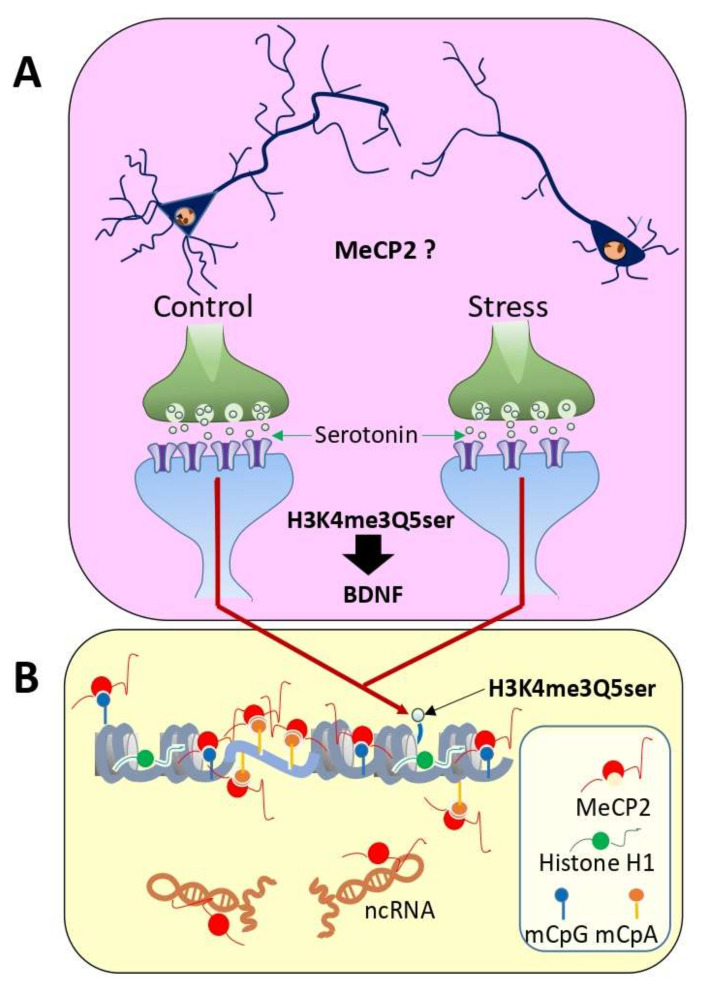
Molecular epigenetic markers with preferential brain specificity: (**A**) schematic representation of the decrease in the number of synapses after a period of chronic stress (depression) [43]. MeCP2 plays an important role in dendritic arborization [39]. Additionally, shown is the histone H3K4me3 serotonylation [37] which appears to regulate the expression of brain genes such as BDNF; (**B**) an important aspect of DNA methylation in the brain in addition to CpG also occurs at CpA [27]. Methylated CpA is preferentially bound by MeCP2 [30], where it displaces nucleosomes from CpA-repeat-binding regions (microsatellites) [32]. MeCP2 is highly abundant in cortical neurons, where it binds with a stoichiometry of approximately one molecule for every two nucleosomes [29]. In addition to methylated DNA, MeCP2 also binds to ncRNAs [46,47] (see [48] for a review).

Figure 1 provides a summary of the unique epigenetic elements of the brain described above, framed within the context of the topic of this review. MeCP2 has been shown to play a crucial role in neuronal dendritic arborization [39,40,41], and chronic stress is known to decrease dendrite length and branching [42] as well as synapse density [43] (Figure 1A). Although the role of serotonin in major depressive disorder (referred to as depression throughout this review) has remained somewhat controversial [44], changes in synaptic serotonin levels and receptor levels have been shown to be coupled with altered synaptic plasticity and neurogenesis [45]. In fact, alterations in serotonin availability might affect the production of H3K4me3Q5 serotonylation within neurons [45], and this histone PTM is involved in promoting a BDNF-dependent neurite outgrowth [37]. In addition, MeCP2 preferentially binds to methylated CpA (Figure 1B) [30]. These observations suggest that more investigation of the potential role of CpA methylation in depression is warranted.

Noncoding RNAs, which can be classified as small (sncRNA, <200 nucleotides in length) or long (lncRNAs, >200 nucleotides in length), have also been shown to contribute to depression [49,50], and MeCP2 is an important ncRNA-binding protein (see [48] for a review). For example, miR-184, an imprinted miRNA whose transcription is repressed by MeCP2 under certain neuronal activity conditions [51] is downregulated in older adults with major depressive disorders [52]. Finally, there is evidence that aberrant H3.3 dynamics in the nucleus accumbens (NAc) (a limbic center for reward and mood) promotes vulnerability to depression [53].

## 3. Stress and Synaptic Plasticity

Stress is described as a state of threatened homeostasis to the body, created by exposure to a variety of different types of stressors. These can range from environmental factors (e.g., deadlines, job loss, sickness, social interactions, noise, weather) to homeostatic challenges (e.g., dehydration, diet, exercise). The ability to cope with stressors relies upon the activation of the hypothalamic–pituitary–adrenal (HPA) axis. The hypothalamus comprises many heterogeneous nuclei that control distinct physiological functions, among which is the paraventricular nucleus (PVN), a main HPA axis regulator [54]. The PVN is activated when stressors are perceived by the body and is responsible for the initiation of a cascade of neuroendocrine events that enable a “flight-or-fight” response to escape from the stress and return to homeostasis [55]. The first step involves the activation of corticotropin-releasing hormone (CRH) in neurons in the parvocellular PVN that project to the anterior pituitary to stimulate the production and secretion of adrenocorticotropic hormone (ACTH) [55,56]. ACTH is then released into the peripheral circulatory system, where it reaches the adrenal gland and stimulates the production and secretion of glucocorticoids (GC). Glucocorticoids are then distributed throughout the body to stimulate glucose metabolism, influence gene expression and immune system activity, etc., to mount a stress response [57,58]. When the stressor ceases, GCs are diminished via inhibitory feedback on the HPA axis, which causes decreased transcriptional activation of proteins involved in the activation of the stress response (i.e., CRH) [54]. As protection against harm, this acute response to a single stressor works remarkably well. However, when chronic stressors occur, the HPA system goes into overdrive and feedback inhibition fails, resulting in abnormal GC levels, which eventually begin to damage homeostatic systems in the body. It is also important to note that there are clear sex differences in the magnitude and length of HPA axis responses to stress [59].

Sex is an important determinant of human health, as evinced by the observation of sex-specific prevalence rates of various mental and physical disorders. There are also sex-specific patterns of HPA axis activation following perceived, or psychological, threats or stressors [60]. Females show a greater hormonal response to stress that provides a higher ability to cope with stress; however, the negative feedback that shuts down the stress response and brings GC levels to baseline is reduced in females, which, in the case of chronic stress, can make them more vulnerable to high levels of GCs. These intrinsic sex differences could underlie differential vulnerabilities that would explain why women are twice as likely as men to develop stress-related disorders such as depression [61,62].

Stress activation in the PVN is also dependent on the nature of the stressor, and it involves a complex system of inhibitory and excitatory projections originating in multiple brain regions, thereby making it a highly plastic nucleus [63,64]. In neurons, plasticity or more precisely synaptic plasticity can be described as the activity-dependent modification of synaptic transmission of pre-existing synapses by the increase in strength or efficacy of their transmission. There are two main types of synaptic plasticity—short-term and long-term plasticity. Synaptic plasticity exists in many forms, lasting from milliseconds to minutes, and plays a critical role in rapid adaptation to sensory input, transient changes in behavior, and short-term memory. Long-term synaptic plasticity, on the other hand, involves an activity-dependent, long-term change in synaptic efficacy that can either enhance (long-term potentiation (LTP)) or depress synaptic strength (long-term depression (LTD)).

Synaptic plasticity is influenced by stress and impairments in synaptic plasticity mechanisms could contribute to stress-related pathology [55,65].

## 4. MeCP2 and Synaptic Plasticity

MeCP2 has been widely studied for its involvement in Rett syndrome (RTT), a neuronal disorder characterized by a loss of function resulting from mutations in the MeCP2 gene that result in neurodevelopmental and cognitive alterations [66]. The association between MeCP2 and synaptic plasticity was first hypothesized from the observations that RTT individuals present alterations in the number and morphology of dendritic spines [67], decreased dendritic growth [68], and spine dysgenesis [69]. All these alterations, together with the increase that takes place in the neuronal expression of MeCP2 during early development in the normal brain [70,71], suggested an important role of this protein in synapse formation and maintenance, which are key to synaptic plasticity.

As the research that can be performed on human postmortem brains is limited, animal models of RTT have been developed and used to further elucidate the role of MeCP2 in brain synaptic plasticity [66,72]. Hippocampal and cortical synaptic dysfunction is observed in several MeCP2-based mouse models of RTT, as well as dendritic growth and morphology alterations [73,74,75]. Loss-of-function mutations in MeCP2 result in atypical structural synaptic plasticity [76,77] and reduced paired-pulse responses and alterations of other parameters of short-term synaptic plasticity [78,79]. Loss of MeCP2 also leads to changes in neuronal excitability and deficits in experience-dependent and activity-dependent refinement [80].

MeCP2 gain of function is associated with MeCP2 duplication syndrome (MDS), which causes severe mental retardation, stunted motor development, early-onset hypotonia, epileptic seizures, and progressive spasticity [81], and interestingly is also associated with mood alterations such as anxiety, depression, and an autistic-like phenotype [82]. In contrast to MeCP2 loss-of-function models, MeCP2 gain-of-function models show rescue of dendritic defects [83] and display augmented paired-pulse facilitation [84,85] indicative of a tight relationship between MeCP2 and synaptic plasticity. Most recent studies have shown that an aberrant increase in the formation and stabilization of dendritic spines occurs in the cerebral cortex of the mouse model of MECP2-duplication syndrome and seems to be mediated by hyperactivation of the ERK pathway [86,87], a major regulator of clustered spine stabilization [88].

Similar effects in synaptic plasticity have also been observed in vitro (for a review, see [89]). For example, mutation of MeCP2 at S421 blocks dendritic growth and spine maturation in an in vitro overexpression model of RTT [90], whereas knockdown of endogenous MeCP2 using a short-hairpin RNA caused a significant reduction in the total spine density in human postmortem hippocampal cultures [91]. Human neurons derived from patients with RTT show a significant deficit in neuron-specific K^+^-Cl^−^ cotransporter 2 expression, a target of MeCP2, and a critical transporter for regulating the polarity and efficacy of GABAergic neurons [92].

**Figure 2:**
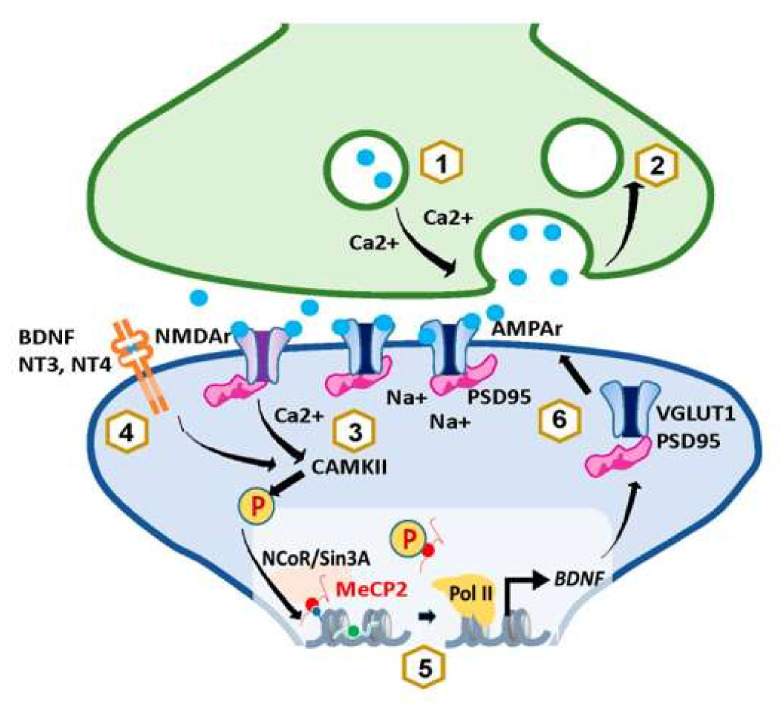
Bidirectional relationship between MeCP2 and short-term plasticity. Evidence shows that MeCP2 increases mEPSC response and frequency, as well as RRP charge, which could be induced by either affecting calcium levels (1) or transcription of presynaptic plasticity proteins involved in vesicle release and trafficking (2) [78,85,101,102]. Moreover, it can influence postsynaptic plasticity by modulating the transcriptional activation of BDNF (5). The activation of BDNF leads to the insertion of NMDA receptors and PSD-95 in the postsynaptic terminal, which increases synaptic strength (6) [109]. Reciprocally, an increase in synaptic activity also influences the levels of MeCP2. For instance, NMDA-receptor-mediated depolarization of the postsynaptic membrane causes an increase in calcium influx that leads to MeCP2 phosphorylation at S421 by CamKII (3) [93]. Stimulation with the neurotrophins BDNF, NT3, and NT4 at the synapse can also trigger CamKII-dependent MeCP2 S421 phosphorylation (4) [90]. Phosphorylation of MeCP2 weakens its interaction with DNA and results in its release from NCoR or Sin3A corepressor complexes allowing DNA polymerase II to initiate the transcription of genes involved in synaptic plasticity such as BDNF (5) [90].

All these studies show that MeCP2 affects synaptic strength and plasticity, but the specific underlying mechanisms are not completely understood. Phosphorylation of MeCP2 at Serine 421(pS421) occurs upon neuronal depolarization and can lead to an activity-dependent gene transcription important for neuronal connectivity and synaptic plasticity in the nervous system. Indeed, results obtained in vitro and in vivo, using a highly specific antibody recognizing this phosphorylation site (pS421) and using transfections with plasmids expressing an S421A mutation, found that such mutation prevents MeCP2 phosphorylation, which, in turn, causes an impaired dendritic growth and spine maturation [90]. It has recently been documented that MeCP2 is primarily phosphorylated at S421 by CamKII, in response to the calcium influx caused by neuronal depolarization during neuronal activity [93]. However, activation of CamKII and further MeCP2 phosphorylation has also been achieved in neuronal cultures stimulated with the brain-derived neurotrophic factor (BDNF), and neurotrophins 3(NT3) and 4 (NT4), suggesting that neurotrophin signaling may regulate MeCP2 function in cooperation with neuronal activity [90] (Figure 2). Interestingly, MeCP2 pS421 has so far only been detected in the brain, where it seems to play a very important role in stimulus-induced neuronal activity. For example, it plays a role in the light-induced activation of neurons in the suprachiasmatic nucleus (SCN) [90]. More importantly, especially in the context of this review, MeCP2 S421 phosphorylation can be induced by early life stress in neurons of the parvocellular hypothalamic PVN [94], the previously described center of stress regulation. Moreover, antidepressant drugs such as imipramine, fluoxetine, or ketamine can induce S421 phosphorylation and reverse stress-induced depression, which will be discussed in the next section of this review [95,96,97].

Evidence regarding the role of MeCP2 in regulating proteins involved in neurotransmitter release also exists. For instance, MeCP2-mutated neurons derived from hiPSCs [98], neurons lacking MeCP2 derived from mice [99], or organoids from RTT patients [89] show alterations in VGLUT1, a specific protein of glutamatergic neurons responsible for loading glutamate into synaptic vesicles. Moreover, a study in the Rett syndrome mouse model revealed alterations in another presynaptic protein, SV2A [100]. There is also plenty of evidence for MeCP2 affecting presynaptic function. MeCP2 increases miniature excitatory postsynaptic current (mEPSC) response and frequency, as well as readily releasable pool (RRP) charge, which could be induced by either altering calcium levels or transcription of presynaptic plasticity genes [78,85,101,102] (Figure 2). As for the effects of MeCP2 in postsynaptic plasticity, studies show that overexpression of MeCP2 causes an increase in glutamatergic synapse number [91]. This increase seems to play a role in mediating homeostatic plasticity and provides a negative feedback mechanism used by neurons to offset excessive excitation or inhibition by adjusting their synaptic strengths [103]. This could be explained by the fact that both calcium or neurotrophin-induced S421 phosphorylation results in the release of MeCP2 from corepressor complexes such as the nuclear receptor corepressor (NCoR) [104] or the switch independent 3 gene-encoded protein A (Sin3A) [105] (Figure 2). This would lead to the transcriptional activation of neuronal modulators that play key roles in neuronal survival, development, and plasticity such as BDNF [90,102,105,106,107,108] (Figure 2). Such mechanisms would suggest mutual functional coregulation of MeCP2 and BDNF.

In summary, there is a wide body of evidence for the involvement of MeCP2 in brain synaptic plasticity, suggesting a bidirectional relationship between short-term plasticity and MeCP2 levels. MeCP2 levels could, either directly affect the calcium concentration in the presynaptic terminal or perhaps may alter proteins involved in neurotransmitter release. Moreover, it can influence postsynaptic plasticity by modulating GABAergic and glutamatergic transmission, leading to alterations in neuronal activity and connectivity. Such alterations could underlie the loss of motor and cognitive abilities and impaired social interactions seen in individuals with RTT, as well as the anxiety and depressive symptoms present in individuals with depression.

## 5. MeCP2 and Stress-Related Pathology: Focus on Depression

Stress-related disorders such as depression share similar synaptic plasticity alterations to those observed in MeCP2 loss- or gain-of-function models [110]. In this section, we summarize the existing data on the putative involvement of MeCP2 in stress-related pathology with a special focus on depression (Table 1).

Early life stress (ELS) typically results in sustained HPA-axis deregulation and is a major risk factor for stress-related diseases, in particular depression. Interestingly, MeCP2 has been shown to contribute to ELS-dependent epigenetic programming of genes that enhance HPA-axis activity [111]. For instance, MeCP2 acts in association with the histone deacetylase 2 (HDAC2) and DNA methyltransferase 1(DNMT1) to repress pro-opiomelanocortin (Pomc—the polypeptide precursor of ACTH) gene expression in an ELS paradigm [112]. There is also evidence that ELS induced by maternal separation (MS), which causes depression-like behavior in rats and can be reversed with antidepressants, is associated with an increase in MeCP2 binding to the exon IV but not exon I of the BDNF promoter in the hippocampus, which elicits a negative regulation of BDNF expression that can be reversed by chronic antidepressant treatment with escitalopram [113,114].

Similar alterations have been observed in other stress paradigms. For example, chronic unpredictable stress (CUS) in rodents causes depression-like behaviors that are accompanied by a decrease in MeCP2 and BDNF levels in the hippocampus, as well as an increase in miR-132, which is negatively correlated with MeCP2 and BDNF levels in blood samples from patients with depression [115]. The same decrease in MeCP2 and an increase in oxidative stress were observed in a study using social defeat as a stressor [116]. However, a study using the chronic social defeat stress (CSDS) model, revealed increased protein levels of MeCP2 in the hippocampus, compared with controls and CSDS-resilient mice. In this study, CSDS animals had reduced protein levels of the transcription nuclear factor-erythroid factor 2-related factor 2 (Nrf2) and BDNF in the medial prefrontal cortex (MPFC) and hippocampus, compared with controls and CSDS-resilient mice. Furthermore, activation of Nrf2 by sulforaphane induced fast-acting antidepressant-like effects in mice potentially by activating BDNF transcription, as well as inhibiting MeCP2 expression, which was accompanied by a decrease in abnormal synaptic transmission [117]. Moreover, it has recently been shown that Nrf2 activation in microglia is associated with an improvement in dendritic spine morphology in stressed mice [118]. Finally, a study looking at hippocampal sections of mice stressed by social isolation supported a role of MeCP2 in modulating oxidative stress [119], which has already been seen in RTT [120,121]. This can be explained by the actions of MeCP2 on genes essential in antioxidant and radical scavenger pathways. Interestingly, oxidative stress contributes to both depression and MeCP2-related disorders (for more details, see [122,123]). This compilation of studies using different stress paradigms provide consistent support for the role of MeCP2 in stress-related pathology.

Further evidence of MeCP2 involvement in stress-related pathologies such as depression is provided by the observation that the therapeutic effects of most antidepressant drugs rely on changes in MeCP2 levels. For example, MeCP2- phosphorylation at Ser421 was recently reported to be essential for the sustained, but not the rapid, antidepressant effects of ketamine and scopolamine in mice [118]. Moreover, the ability of imipramine to reverse depression-like behaviors induced by chronic social defeat stress involves MeCP2 phosphorylation [123] and alterations in BDNF and MeCP2 caused by CUS are normalized by escitalopram [124]. Fluoxetine treatment, which improves depressive-like behavior, also dissociates the MeCP2–CREB complex from BDNF promoter IV by protein kinase A (PKA)-mediated phosphorylation of MeCP2 at Ser421, increasing BDNF transcription resulting [107]. Finally, a recent study using a chronic social defeat stress model reported that the fast-acting antidepressant effects of (R)-ketamine, the enantiomer of the FDA-approved antidepressant (S)-ketamine, was mediated by the increased BDNF transcription. Such an increase resulted from the activation of CREB and MeCP2 suppression in microglia [97].

Although these observations in animal models are exciting, it is important to acknowledge that they may not translate to the human condition. There is almost no literature looking at alterations in MeCP2 levels in depressed patients. We have only been able to find two studies that assessed this: An early study examined blood samples of individuals suffering from depression and found a negative correlation between MeCP2 and miR-132 expression levels, which is a microRNA associated with depressive-like behavior [115]. A more recent study examined postmortem brains of individuals who died by suicide and found a decreased BDNF and p-S421p-MeCP2/MeCP2 protein ratio, compared with controls. However, whether these individuals were suffering from depression was not reported [125]. Nevertheless, the results from these two studies are consistent with those from more extensive studies using animal models that we just described.

Altogether, these data indicate that MeCP2 may play a key role in early and adult life stress-induced epigenetic modifications that lead to the development of depression-like behaviors via MeCP2-dependent transcriptional regulation. This might provide more insight into the mechanisms of antidepressant drug action and even provide a new target for novel antidepressants. More clinical studies that focus on examining changes in MeCP2 and other markers of epigenetic change in patients with depression are badly needed.

**Table 1:** MeCP2 involvement in stress-related pathology, focusing on depressive symptoms.

Subject	Experimental Paradigm	MeCP2 Involvement	References
Mice	MeCP2 knock-in and chronic social defeat stress (CSDS) Imipramine treatment	pMeCP2 is required for the effects of chronic imipramine on depressive-like behaviors induced by chronic social defeat stress.	[123]
	Early life stress (ELS) by maternal separation (MS)	They suggest that MeCP2 acts in association with the chromatin modifiers HDAC2 and DNMT1, to repress Pomc gene expression in the ELS paradigm.	[112]
	Post-stroke depression (PSD) fluoxetine treatment(FLX)	Fluoxetine improved depression-like behaviors of PSD mice and upregulated the expression of BDNF in the hippocampus but depletion of BDNF suppressed the effect of fluoxetine. FLX treatment also disassociated the MeCP2-CREB-Bdnf promoter IV complex via phosphorylation of MeCP2 at Ser421 by PKA.	[96]
	Social isolation stress (SI)	Decreased PPAR-α expression in the hippocampus of SI mice was associated with increased MeCP2, which favored hypermethylation and was also associated with increased TLR-4 and pro-inflammatory markers, mediated by NF-κB signaling in the hippocampus of aggressive mice.	[119]
	Chronic social defeat stress	Nrf2 induces BDNF transcription via upregulation of Nrf2 and downregulation of MeCP2 in microglia, which is associated with changes in the morphology of damaged dendritic spines in stressed mice.	[117]
	Chronic social defeat stress	Activation of Nrf2 by sulforaphane showed fast-acting antidepressant-like effects in mice by activating BDNF and inhibiting MeCP2 but not in Nrf2 knockout mice. In contrast, levels of MeCP2 in the CSDS-susceptible mice were higher than those of control and CSDS-resilient mice.	[126]
	Chronic social defeat stress (R)-ketamine treatment	(R)-ketamine fast-acting antidepressant effects are suggested to be mediated by an increase in BDNF transcription induced by the activation of CREB and MeCP2 suppression in microglia in a CSDS model of Depression.	[97]
Rats	Chronic unpredictable stress (CUS) rat model of depression	Knockdown of MeCP2 expression in primary hippocampal neurons increased miR-132 expression and decreased BDNF expression. CUS-induced depressive-like behaviors correlated with an increase in miR-132 and decreased levels of MeCP2 and BDNF in the hippocampus.	[115]
	Social defeat stress (SD) Exercise treatment	Moderate exercise rescued social defeat induced anxiety-like behavior and memory impairment, and normalized SD-induced increase in oxidative stress, leading to decreased MeCP2 protein levels in the hippocampus.	[116]
	Early life stress by maternal separation and adult restraint stress (RS). Escitalopram(ESC) treatment.	Both the MS and RS groups had increased MeCP2 levels at hippocampal BDNF promoter IV with a greater effect by combining MS and RS. This was associated with an increased despair-like behavior measured with the forced swim test. Chronic escitalopram treatment recovered these alterations.	[113]
	Early life stress by maternal separation (MS). Escitalopram treatment.	Escitalopram treatment decreased MeCP2 binding to the BDNF promoter exon I in the hippocampus of MS animals; however, MS had no effects on MeCP2 binding levels, compared with controls. MS animals treated with ESC revealed significant increases in BDNF protein and significant decreases in MeCP2 mRNA levels.	[114]
	Chronic unpredictable mild stress (CUMS) rat model of depression. Escitalopram treatment.	CUMS increased depressive-like behavior but did not change MeCP2 expression, compared with controls. CUMS reduced BDNF levels in the hippocampus and increased them in the frontal lobe. ESC increased BDNF levels in the hippocampus and increased MeCP2 levels in the hippocampus and the frontal lobe.	[124]
Humans	Major Depression	MeCP2 and BDNF negatively correlated with miR-132 expression levels in the blood of depression patients.	[115]
	Nondiagnosed psychiatric suicide	Suicide samples have decreased BDNF, increased H3K27me2, Sin3a levels, and decreased p-S421-MeCP2/ MeCP2 protein ratio. They suggest a role in MeCP2 in lowering BDNF protein levels in suicide patients.	[125]

Abbreviations: MeCP2, methyl-CpG-binding protein 2; HDAC2, histone deacetylase 2; DNMT1, DNA methyltransferase 1; Pomc, pro-opiomelanocortin; BDNF, brain-derived neurotrophic factor; TLR-4, Toll-like receptor 4; PPAR-α, peroxisome proliferator-activated receptor alpha; PKA, protein kinase A; Nrf2, nuclear factor erythroid 2-related factor 2; miR-132, microRNA-132; H3K27me2, histone 3 lysine 27 dimethylation.

## 6. MeCP2 and Reelin

Reelin is a pleiotropic extracellular matrix protein that regulates neural migration and maturation, and dendritic sprouting during brain development, and is involved in neural plasticity in the adult brain [127,128,129]. Expression of the reelin gene (RELN) is downregulated in multiple psychiatric disorders, including a decrease in reelin expression levels in the CA4 hippocampal region in major depressive disorder [130]. Similar findings have been observed in animal models of chronic stress, where reelin expression is downregulated in the dentate gyrus subgranular zone and is rescued by antidepressant treatment [131,132]. In fact, intrahippocampal infusions of recombinant reelin rescue the behavioral deficits associated with chronic stress in the repeated-corticosterone paradigm [133]. Investigations on the origin of reelin downregulation in multiple psychiatric disorders prompted the investigation of epigenetic mechanisms, regulating reelin expression (see below).

The human RELN promoter can bind several transcription factors such as Sp1 and CREB and provides several susceptible methylation sites (CpGs) [134,135,136]. As previously described, MeCP2 can bind to mCpG sites in promoters; therefore, it is not surprising to find that MeCP2 binds to hypermethylated RELN promoter suppresses its transcription by recruiting a local repressor complex [137,138]. HDAC inhibitors suppress the actions of histone deacetylases, which can then disrupt repressor complexes such as the one in the RELN promoter, thus leading to an increase in reelin levels. This is interesting because HDAC inhibitors can reverse stress-induced depression-like symptoms in rodents in a similar way to current antidepressants [139], which brings more evidence supporting the role of reelin in the pathophysiology of depression.

Human and animal studies of bipolar, autism spectrum disorder (ASD), and schizophrenia have revealed the presence of hypermethylated RELN promoters, which correlate with a decrease in reelin protein levels [137,140,141,142]. Moreover, overexpression of the methyl-transferases DNMT1(maintenance methylation) and DNMT3a (de novo methylation) in GABAergic interneurons, which express high levels of reelin, is present in bipolar and schizophrenia and is associated with alterations in mood and synaptic plasticity [143,144,145,146]. In addition, prenatal stress in rodents increases binding of DNMT1 and MeCP2 and increases 5-methylcytosine and 5-hydroxymethylcytosine in specific CpG-rich regions of the RELN and GAD67 promoters. This is associated with alterations in schizophrenia-like behaviors that are corrected with the administration of a histone deacetylase inhibitor (valproic acid) or an atypical antipsychotic with DNA–demethylation activity (clozapine) [147]. Similarly, in postmortem brains of patients with ASD, there is increased binding of MeCP2 to the GAD1 and RELN promoters in the cerebellum, probably mediated by an enrichment of 5-hmC [148]. All these studies support the dysregulation of reelin to be associated with an epigenetic mechanism in several psychiatric disorders that have many commonalities with depression.

**Figure 3:**
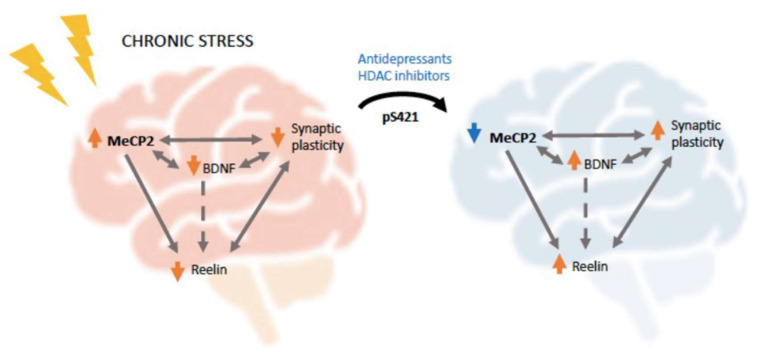
Schematic representation of the putative ways MeCP2 could be contributing to stress-related pathology. Chronic stress instigates an increase in MeCP2 levels, which leads to its increased binding to gene promotors such as BDNF or RELN, inducing their transcriptional repression. MeCP2, BDNF, and reelin all contribute to promoting synaptic strength and neurotransmission; thus, stress-induced alterations in these proteins contribute to a decrease in synaptic plasticity [83,84,85,149]. Moreover, BDNF could also be modulating reelin levels through changes in its receptors [46]. Different SSRIs and ketamine induce the phosphorylation of Ser421 residue in MeCP2, which seem to be necessary for their antidepressant effects [96,118]. Similarly, HDAC inhibitors, which also show antidepressant effects, disrupt the MeCP2 repressor complex in the RELN promoter [140]. BDNF can also participate in this phosphorylation creating a reciprocal relationship with MeCP2 [90]. Finally, a decrease in MeCP2 repressor activity will lead to an increase in transcriptional activation of BDNF and RELN, leading to the restoration of synaptic plasticity and the alleviation of depressive-like symptomatology.

Finally, although we demonstrated that stress alters MeCP2 levels and that RELN expression is epigenetically regulated by MeCP2, most of the literature only focuses on the effects of MeCP2 in modulating BDNF. Even though BDNF can also modulate reelin receptor levels [149] (Figure 3) and thus be an indirect link between MeCP2 and reelin, we hypothesize that there might be an interesting direct link between the two supported by the deficient MeCP2 and RELN signaling observed in multiple psychiatric conditions, as well as the related effects observed with BDNF, which itself modulates RELN and synaptic plasticity (Figure 3). We recognize that this association, described in Figure 3, is still speculative due to the lack of substantial evidence; nevertheless, it represents a very interesting research gap worth investigating in the future.

Altogether, in this study, we described the known functions of MeCP2 as an epigenetic regulator, and we provided evidence for its role in modulating synaptic plasticity via the transcriptional regulation of BDNF or other proteins involved in synaptogenesis and synaptic strength like reelin. We also revealed that environmental factors such as chronic stress cause the development of a depressive phenotype that can be reversed with common antidepressants and parallel with alterations in MeCP2 levels. Chronic stress is a potent risk factor for depression, and patients with depression show abnormal synaptic plasticity and reelin levels, which, according to the evidence gathered here, seem to be mediated, in part, by the epigenetic actions of MeCP2. Given the clear need for better treatment options for depression, as up to 50% of patients do not respond to current antidepressants, we believe additional studies of the role of MeCP2 in depression are necessary and may open new opportunities for the development of novel antidepressants.

## Data Availability

Not applicable.

## Abbreviations

ACTH, adrenocorticotropic hormone; BDNF, brain-derived neurotrophic factor; CaMKII, Ca2+/calmodulin-dependent protein kinase II; CREB, cAMP response element-binding protein; CRH, corticotropin-releasing hormone; CSDS, chronic social defeat stress; CUS, chronic unpredictable stress; ELS, early life stress; Fkbp5, FK506-binding protein 5; GC, glucocorticoids; HFD, histone fold domain; HPA, hypothalamic–pituitary–adrenal; IDD, intrinsically disordered domain; lncRNA, long noncoding RNA; LTD/P, long-term depression/potentiation; MBD, methyl-binding domain; MeCP, methyl-CpG-binding protein; MDD, major depressive disorder; MDS, MeCP2 duplication syndrome; mEPSC, miniature excitatory postsynaptic currents; MPFC, medial prefrontal cortex; MS, maternal separation, NAc, nucleus accumbens; NCoR, nuclear receptor corepressor; ncRNA, noncoding RNA; NMDA, N-Methyl-d-aspartate; Nrf2,nuclear factor-erythroid factor 2-related factor 2; NT3/4, neurotrophin 3/4; PKA, protein kinase A; PSD-95, postsynaptic density protein 95; PTM, post-translational modification; Pomc, pro-opiomelanocortin; PVN, paraventricular nucleus; RRP, readily releasable pool; RTT, Rett syndrome; Sgk1, serum glucocorticoid-inducible kinase 1; SCN, suprachiasmatic nucleus; Sin3A, switch independent 3 gene-encoded protein A; WHD, winged helix domain.
